# Computation of Equilibrium Bilayer Monodisperse Foam Structures Using the Surface Evolver

**DOI:** 10.1038/s41598-017-05490-y

**Published:** 2017-07-21

**Authors:** Fuyang Li, Chengchuan Zhang, K. A. Brakke, Zuosheng Lei

**Affiliations:** 1 State Key Laboratory of Advanced Special Steel & Shanghai Key Laboratory of Advanced Ferro-metallurgy & School of Materials Science and Engineering, Shanghai University, Shanghai, 200072 China; 2 Mathematics Department, Susquehanna University, Selinsgrove, PA 17870 USA

## Abstract

The Surface Evolver is used to minimize the surface energy of two ordered structures for bilayer monodisperse wet foams with arbitrary liquid fraction. Previous researchers have found a reversible structural transition in bilayer monodisperse foams by changing the foam liquid fraction in a physical experiment. We simulated this phenomenon by analyzing the interfacial energy of two bilayer foam systems with varying liquid fractions. The calculations reported here show that the Tóth structure is energy minimizing when the liquid fraction is below a critical value, around 2.26%, above which point the honeycomb structure becomes preferable, although the Tóth structure remains metastable.

## Introduction

**Figure 1:**
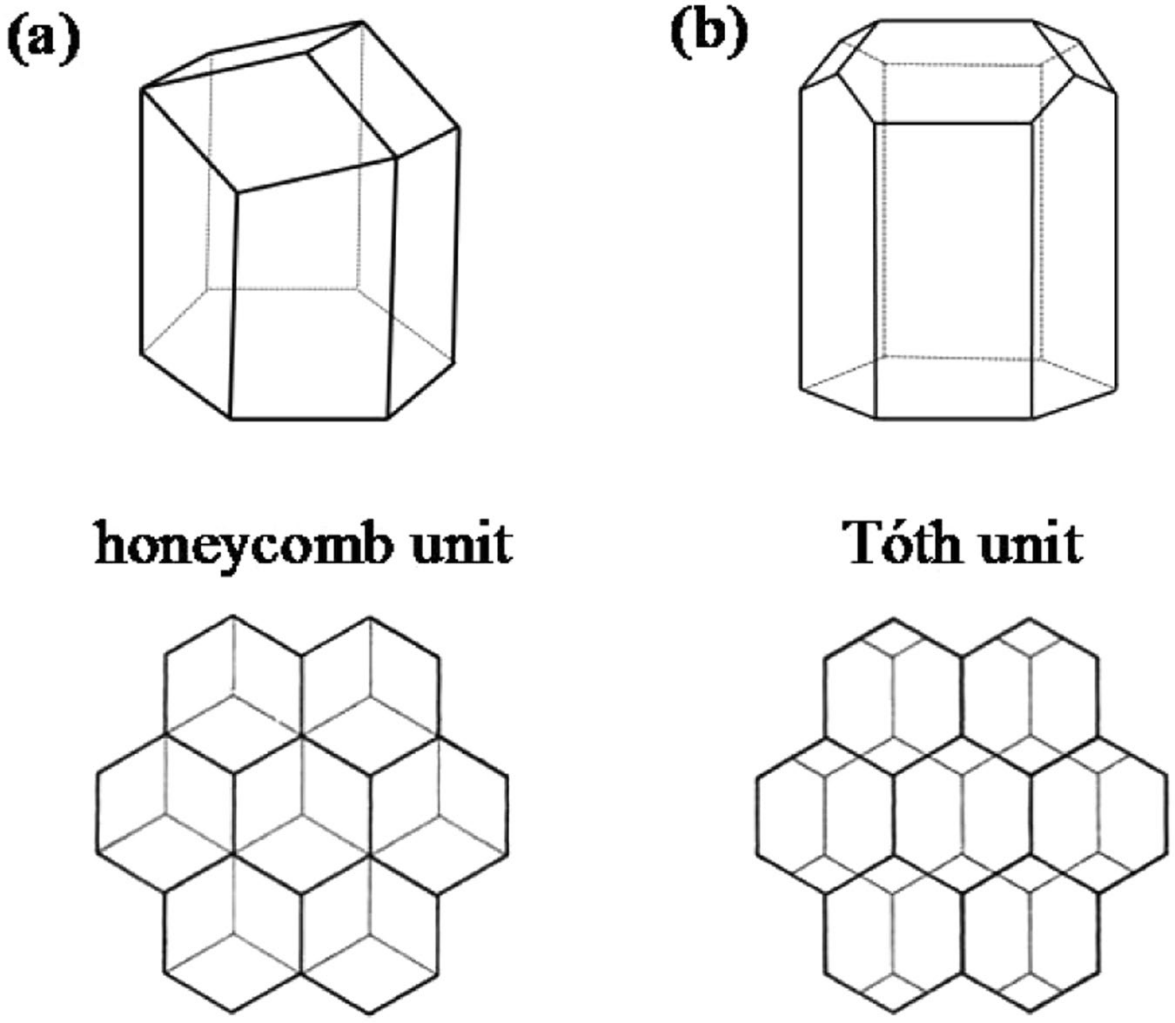
(**a**) A honeycomb unit and a view of such units projected on a plane; (**b**) A Tóth unit and a view of such units projected on a plane.

Bilayer monodisperse foams are highly structured fluid configurations in which series of equal-sized bubbles are dispersed between two parallel flat boundaries. Each layer forms an array of hexagonal cells. The foam may be “dry,” an ideal state with no liquid within the foam surfaces, or “wet,” with liquid in “Plateau borders” where three surfaces meet. In terms of minimizing the energy of the cell structure, there are two competing configurations in the system of dry bilayer monodisperse foams. One is the honeycomb structure, composed of a series of honeycomb units (Fig. 1a), which was once believed to have minimal surface area with given volume^1, 2^. The other structure is composed of a series of Tóth units (Fig. 1b), which was discovered by L. F. Tóth^3^ in 1964, and now named the Tóth structure. For dry foams, it proved to have 0.35% less surface area than the honeycomb unit. In 1994, Weaire and Phelan^4^ found these two structures in an experiment, and stated that a foam of low liquid content corresponds to the Tóth structure. When adding more liquid to the low liquid content foam, the Tóth structure switched to the honeycomb structure.

Researchers believe the structural transition described above is a product of foam self-organization for minimizing system energy. So far, foam structural transition is mostly obtained by changing the liquid fraction, which is the volume fraction of liquid content. If we can introduce numerical simulation and accurately analyze how the energy of these bilayer foam systems changes with liquid fractions and geometric parameters, it would be informative to transition mechanism research.

Our simulations provide convincing evidence that under the same geometrical conditions, the two energy curves as a function of liquid fraction must cross somewhere. In addition, two new concepts, Tóth-like structures and honeycomb-like structures, are introduced. The Tóth-like structure is characterized by a cell contacting four cells in the other layer, and the honeycomb-like structure is characterized by a cell contacting three cells in the other layer. Based on this, the phase diagram for Tóth-like and honeycomb-like structures are discussed in this paper.

A foam consists of gas cells (bubbles) in a liquid medium, separated by thin films wherever the cells impinge on one another. Depending on their size and the pressure of the liquid in their interstices, the bubbles may remain approximately spherical with little contact (wet foam) or be pressed together to form polyhedra with possibly curved faces (dry foam). Several idealizations are commonly applied to describe such a system in simple mathematical terms^5^:

- The thickness of the thin films is neglected.
- The bubble surfaces are associated with a constant surface energy per unit area. No other energy terms are considered, in particular, no gravity.
- Gas and liquid are both incompressible.

The liquid content is contained in two regions^6^: the thin foam films that separate adjacent bubbles, and the Plateau borders where several films meet. The Plateau borders form an interconnected network of channels. The topology and geometry of a dry foam system^7^ were described by Plateau in 1873 using what are now named Plateau’s rules: each film has uniform mean curvature; three films meet symmetrically in a Plateau border at equal angles of 120°; and four Plateau borders meet symmetrically in a vertex at the tetrahedral angle of cos^−1^(−1/3) ≈ 109.47°.

In terms of the structures of bilayer monodisperse foams, the Tóth structure and the honeycomb structure are composed of series of Tóth units and honeycomb units respectively. Each layer forms an array of hexagonal units, and the upper-layer units are inverted above the lower-layer units. The Tóth unit and honeycomb unit are as shown in Fig. 1.

Note that while each layer is hexagonal in both configurations, the position of the top layer with respect to the bottom is different. In the honeycomb structure, the center of a top cell is above a triple junction of the lower layer, while in the Tóth structure the center of a top cell is above the middle of a horizontal edge of the lower layer.

The perfect hexagonal honeycomb is made with flat films; it so happens they can fit together and obey Plateau’s rule of meeting at 120° angles. But that is not true for the Tóth structure; to minimize its energy requires a slight curvature in its films. The effect of the curvature is small, lowering the energy by about one part in a thousand, but it is of the same order of magnitude as the energy differences between the honeycomb and Tóth structures, so it must be taken into account.

Besides changes in liquid fraction, this paper considers changes in the geometric configuration of the layers. Due to its high symmetry, the honeycomb structure may be expected to have its lowest energy when the layers are regular hexagons and the layer stack exactly as shown in Fig. 1(a). But the Tóth structure does not have as much symmetry; it is possible that a shift in the horizontal aspect ratio may lower the energy. The Surface Evolver^8^ model includes a parameter λ for this aspect ratio, with λ > 1 corresponding to a stretching in the horizontal direction in Fig. 1(b) lower, with a corresponding shrinkage in the vertical direction to keep the horizontal area of the unit cell constant.

Also, for investigating the transition between honeycomb and Tóth structures, the relative offset between the layers can be interpolated between the honeycomb and Tóth structures by means of a parameter α, with α = 0 being Tóth and α = 1 being honeycomb.

The distance between the horizontal plates is denoted by *H*, and the liquid fraction is denoted by *φ*_*liq*_. See the Methods section for a fuller discussion of the parameters.

In this paper, the Surface Evolver software^8^ is used to model configurations for bilayer foam systems and accurately calculate the honeycomb structure and Tóth structure system energies as the liquid fraction is varied. The models each consist of one lower cell and one upper cell, with periodic boundary conditions in the horizontal directions, and 90° contact angles on the upper and lower plates. This unit is displayed in multiple copies in the illustrations.

Two Surface Evolver models were created, one for the honeycomb structure, and one for the Tóth structure, each with Plateau borders. The same evolution script was used for both models and for all the variations of the liquid fractions and geometric parameters. The evolution script appears to give energies accurate to about six digits.

After some initial experimentation to determine relevant ranges of parameters, a grid of cases was run, with liquid fraction varying from 0.001 to 0.030 by 0.001, the interpolation parameter α varying from 0 to 1 by 0.1, the aspect ratio λ varying from 1.000 to 1.050 by 0.002, and the height *H* set at 2.00. The hexagon side length is 1, for the basic honeycomb structure. Some key cases were also run at different heights *H*.

The evolution script also checked for conversion between the two types of structures. The conversion from honeycomb to Tóth is signaled by two opposite faces of a Plateau junction touching to form a new interface between cells (with a margin of error to make sure the contact is not just due to the finiteness of the mesh), and the conversion from Tóth to honeycomb is signaled by the disappearance of an interface.

More details and images can be found in the Methods section at the end of this paper.

## Results

Table 1 lists the properties of the structures for dry foams for *H* = 2.0. In terms of surface energy, the Tóth structure attains its minimum energy when λ ≈ 1.03, and its energy is approximately 0.83% less than the honeycomb structure for the same λ. The perfect Tóth structure, λ = 1, is slightly higher energy than the Tóth-like structure for λ = 1.03, and has approximately 0.49% less energy than the honeycomb structure under the same conditions. Let us note that the area of hexagonal contour is a constant (√3/2) and the perimeter of hexagonal contour changes slightly with λ, rather than being a fixed perimeter.

**Table 1:** The results of bilayer monodisperse systems for the dry foams, for H = 2.00.

Configurations	Liquid fraction	Cell Volume	Area (Surface energy)
Tóth-like structure(λ = 1.03)	0	$\sqrt{3}$	4.159670
Tóth structure	0	$\sqrt{3}$	4.164370
Honeycomb structure	0	$\sqrt{3}$	4.171208

**Figure 2:**
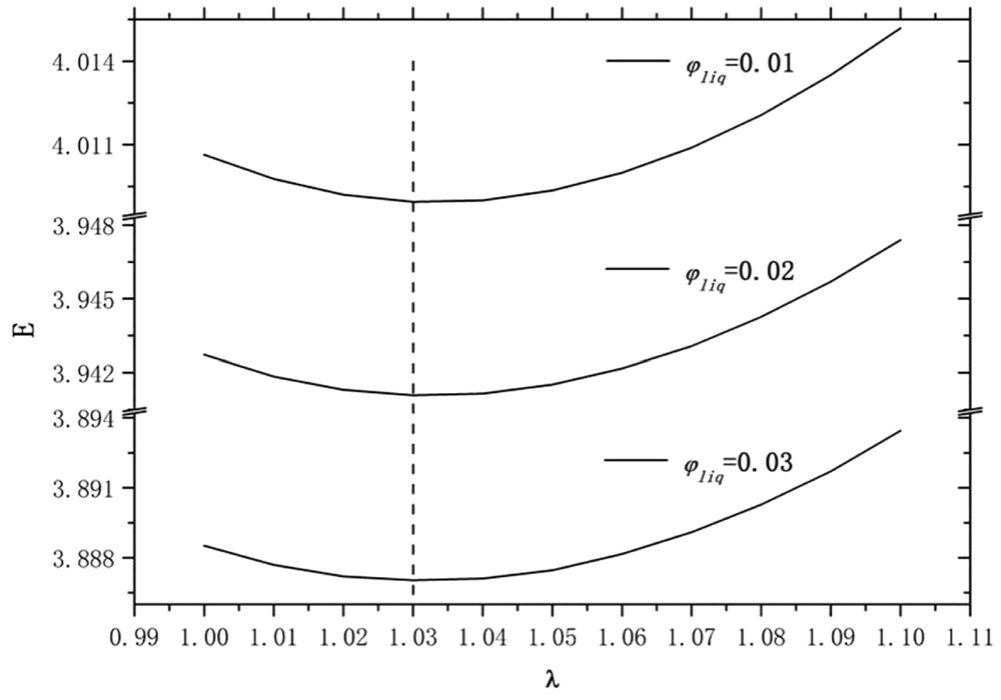
The Tóth structure energy plotted with parameter λ from 1.00 to 1.10 for liquid fractions 0.01, 0.02 and 0.03. The Tóth structure attains its minimum energy for each *φ*_*liq*_ when λ ≈ 1.03.

**Figure 3:**
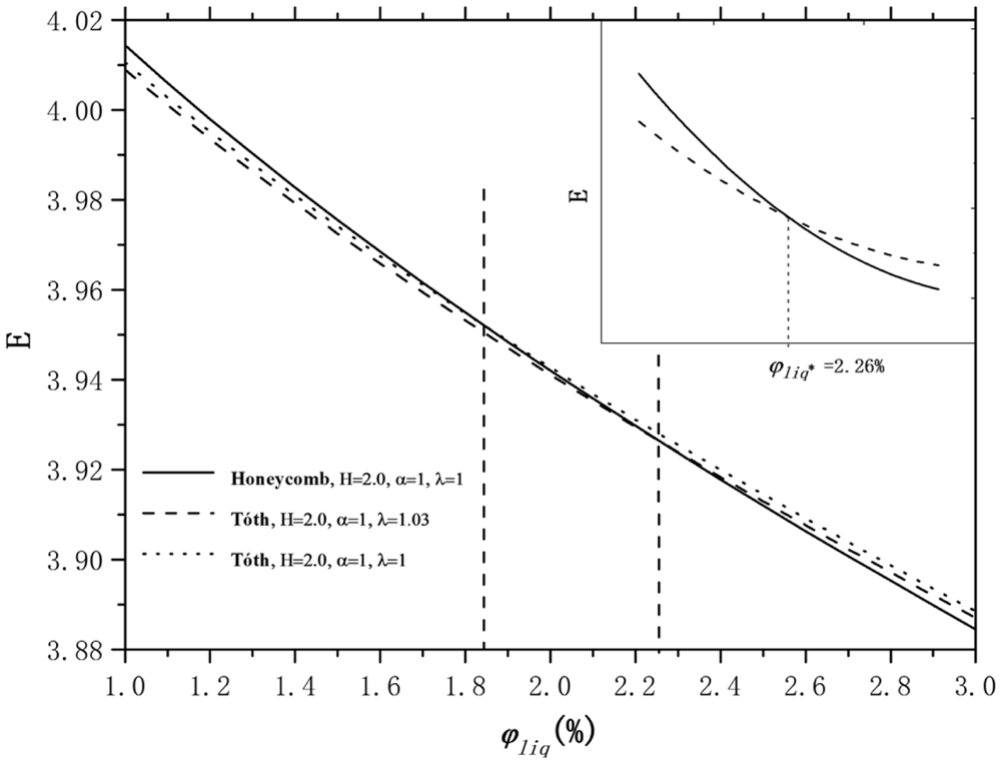
The dimensionless interfacial energy E of the honeycomb structure and the Tóth structure for λ = 1.03 and λ = 1, plotted as a function of liquid fraction *φ*_*liq*_ when *H* = 2.0. The Tóth structure energies include λ = 1, since the external constraints might force λ to be 1. These diagrams show the honeycomb structure and Tóth structure (for λ = 1.03) crossover at about *φ*_*liq*_ = 2.26%, denoted *φ*_liq_^*^. For *φ*_*liq*_ below *φ*_liq_^*^, the Tóth structure has a lower energy. The energies are accurate to about six digits; the critical liquid fraction is probably accurate to at least two digits.

For the wet honeycomb structure, the minimum energy is always at perfect hexagonal symmetry. But the wet Tóth structure attains minimum energy for liquid fractions in the range of 0 < *φ*_*liq*_ < 3% investigated in this paper when λ ≈ 1.03, as shown in Fig. 2. This is found by looking at the grid of results; we have no further mathematical or physical justification. The only reason we can put forward is that λ = 1.03 is best for dry foam, and the liquid Plateau borders do not perturb the surface enough to change that noticeably in our investigated range of liquid fractions. Liquid fractions outside this range this will require more research in future work. Figure 3 plots the computed energies of the Tóth structure for λ = 1.03 and λ = 1, and the honeycomb structure for a range of liquid fractions. We include λ = 1 since external conditions may constrain the freedom to change shape. Because the honeycomb structure and the Tóth structure are very close in energy, it is important to take extreme care in the evolution to get accurate results to at least six decimal places.

We find that under these conditions, the interfacial energy of these two structures (*H* = 2.0) cross at *φ*_liq_^*^ = 2.26%. For *φ*_*liq*_ below *φ*_liq_^*^ = 2.26%, the honeycomb structure foam has a higher energy than the Tóth structure. And for *φ*_*liq*_ above *φ*_liq_^*^ = 2.26%, as the foam becomes wetter, the honeycomb structure has a lower energy, that is, the honeycomb structure is more stable. However, we cannot conclude that at this critical liquid fraction that the Tóth structure becomes unstable and there is a sudden structural transition to honeycomb. The relative positions of the layers are not the same in the two configurations, and there is an energy barrier to get over to make the transition. This was found and reported by Weaire and Phelan^4^ in an experiment. Also, external boundary conditions may inhibit the transition. Hence, metastable states may exist. At some higher liquid fraction, the energy barrier may disappear. However, comparing the energies of honeycomb and Tóth structures is not sufficient to interpret this structural transition; the dynamics of this transition at the critical liquid fraction point cannot be simulated using the Surface Evolver. This will require more transition mechanism research in future work. However, some insight can be gained from inspecting the gradient of energy with respect to α and λ, as will be discussed below.

Results show these two energy curves must cross at a certain liquid fraction discussed above, depending on the height *H* between the horizontal planes. The height used above is *H* = 2.0 and the crossover liquid fraction is about 2.26%. We also calculate the crossover liquid fraction for some different heights to see if height makes any difference.

**Figure 4:**
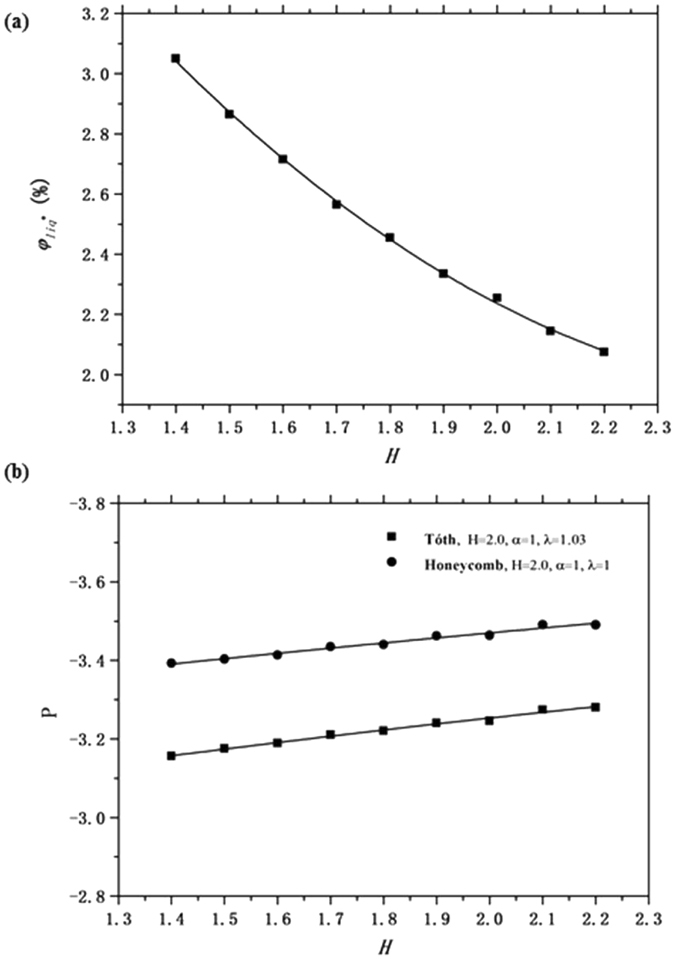
(**a**) The crossover liquid fraction *φ*_liq_^*^ plotted as a function of the height *H* between the horizontal planes. (**b**) The Plateau border pressure P at the crossover liquid fractions plotted as a function of the height *H*.

As shown in Fig. 4(a), we can conclude that the crossover liquid fraction depends on height and the crossover liquid fraction decreases with the increasing height as a whole. The explanation for this is likely that the key factor is the shape of the Plateau borders and junctions where the layers meet, and this shape depends more directly on the pressure in the Plateau borders than on the volume fraction. At constant pressure, changing the height between the horizontal plates merely changes the lengths of the vertical Plateau borders running to the plates. Since the Plateau borders and junctions along the midplane have a higher relative volume fraction than the vertical borders, increasing the height *H* dilutes the impact of the central Plateau borders, and decreases the critical liquid density. Figure 4(b) shows a plot of the critical Plateau border pressure, and shows it to vary by only 3% over the range of liquid fractions covered.

**Figure 5:**
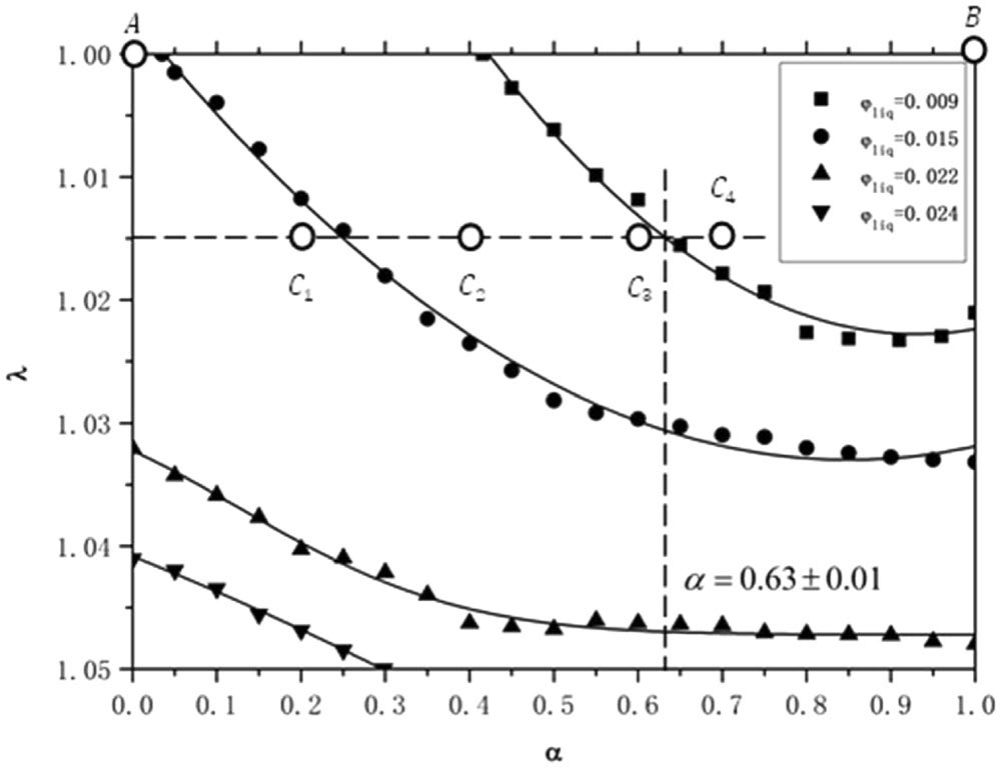
Critical points in α and λ fitted into four curves for four fixed *φ*_*liq*_ in a phase diagram, showing phase transition from honeycomb-like to Tóth-like structures. Six points (*A*, *B*, *C*_*1*_, *C*_*2*_, *C*_*3*_, *C*_*4*_), correspond to six structures as shown in Table 2. There are no curves for Tóth-like converting to honeycomb-like, since such transitions were not found.

In the following section, we consider transitions between the two types of structure. A phase diagram in α and λ for fixed liquid fractions has been made using the Surface Evolver, showing phase transitions between Tóth-like and honeycomb-like structures. As described in the Methods section, the Surface Evolver tests the models for the appearance or disappearance of interfaces. We evolved the models at different points (α,λ) for various fixed *φ*_*liq*_ and *H* = 2.0. The phase diagram is for *H* = 2.0, and we did not do extensive modeling for other heights, because we believe the effect of other bilayer height *H* on the phase diagram is similar to *H* = 2.0. No new phenomena were seen at different heights in the modeling that we did do. To some extent, the phase boundaries may be changed with different heights. But its trend will not change. In addition, doing extensive modeling about other heights requires a great amount of computation. So we just take *H* = 2.0 as an example to study. If there is not a transition detected, the current configuration is stable and exists as a local minimum of energy (perhaps metastable). However, if the Surface Evolver detects a transition then the current configuration is unstable and shifts to the other form of structure. In sum, there were no observed transitions from Tóth-like to honeycomb-like structures over the range of parameters investigated in this paper, but there were transitions from honeycomb-like to Tóth-like. Figure 5 plots critical curves for four sample liquid fractions, *φ*_*liq*_ = 0.009, *φ*_*liq*_ = 0.015, *φ*_*liq*_ = 0.022, and *φ*_*liq*_ = 0.024.

In Fig. 5, the left side of the graph represents perfect Tóth alignment (α = 0) and the right side represents perfect honeycomb alignment (α = 1). A honeycomb-like structure is stable to the right and above the curves, but converts to a Tóth-like structure left and below the curves. A Tóth-like structure is always stable over the range of parameters investigated in this paper. As we stated before, there were no observed transitions from Tóth-like to honeycomb-like structures in this paper. According to this phase diagram, we can conclude the honeycomb-like structure becomes preferable in terms of lower energy as *φ*_*liq*_ is increased, but the Tóth structure will remain metastable and not spontaneously convert.

We take λ = 1.015 as an example to explain the phase diagram in more detail, since the curves have nice slopes there. Table 2 lists the shapes and parameters of six points (*A*, *B*, *C*_*1*_, *C*_*2*_, *C*_*3*_, *C*_*4*_). We find that *C*_*1*_, *C*_*2*_ and *C*_*3*_ are unstable as honeycomb-like structures when *φ*_*liq*_ = 0.009, and convert to Tóth-like structures. However, *C*_*4*_ exists as a stable honeycomb-like structure. Therefore, *C*_*4*_ exists stably as both structures when λ = 1.015, as do other points when α ≥ 0.63 ± 0.01 for λ = 1.015. Hence there are metastable states. When *φ*_*liq*_ = 0.015, both *C*_*2*_ and *C*_*3*_ are stable as honeycomb-like structures. Meanwhile, a *C*_*1*_ honeycomb-like structure converts to a Tóth-like structure because this point is on the left side of the critical line for *φ*_*liq*_ = 0.015. When *φ*_*liq*_ = 0.022 and *φ*_*liq*_ = 0.024, then *C*_*1*_, *C*_*2*_, *C*_*3*_ and *C*_*4*_ all are stable as both kinds of structures since they are above the critical line for *φ*_*liq*_ = 0.022 and *φ*_*liq*_ = 0.024.

**Table 2:** The shapes and parameters of six points (*A*, *B*, *C*_*1*_, *C*_*2*,_ *C*_*3*,_ *C*_*4*_).

Number	The shape of configurations’ top view	α	*λ*	Note
*A*		0	1	Perfect Tóth structure
*B*		1	1	Perfect honeycomb structure
*C*_*1*_		0.2	1.015	Tóth-like structure
*C*_*2*_		0.4	1.015	Tóth-like structure
*C*_*3*_		0.6	1.015	Tóth-like structure
*C*_*4*_		≥ 0.63 ± 0.01	1.015	Honeycomb-like structure

At λ = 1.03, one can see that the Tóth-like structure occurs all the way to α = 1.0 for liquid fractions below about 0.015, but the honeycomb structure persists all the way to α = 0.0 for liquid fraction above about 0.022.

## Discussion

Our simulation has explored the variation of Tóth structure and honeycomb structure with varying liquid fractions and compared the energy of these two structures at different liquid fractions to analyze the conduct of foam self-organization for minimizing system energy. Moreover, based on the Tóth-like structures and honeycomb-like structures, a phase diagram has been made for showing phase transitions between honeycomb-like and Tóth-like structures.

The results show that the Tóth structure attains its minimum energy for each *φ*_*liq*_ when λ ≈ 1.03. Under the same conditions, comparing the lowest energy configurations of honeycomb structure and Tóth structure (λ = 1.03), the interfacial energy of these two structures cross at *φ*_liq_^*^ = 2.26% when the height of bilayer foam system is *H* = 2.0. For *φ*_*liq*_ below *φ*_liq_^*^, the Tóth structure has a lower energy. For *φ*_*liq*_ above *φ*_liq_^*^, the honeycomb structure becomes preferable.

We also found the crossover liquid fraction *φ*_liq_^*^changed with the height *H* of bilayer foam system, and decreased with the increased height as a whole.

In addition, a phase diagram in α and λ for several fixed liquid fractions was made, showing phase transitions between Tóth-like and honeycomb-like structures. The Tóth-like structure is always stable, for the range of parameters covered in this paper. The phase diagram shows that honeycomb-like structure becomes more stable as *φ*_*liq*_ is increased. We expect that the present work will be useful as a basis of future studies relating the energy of bilayer monodisperse foams to their mechanical behavior as well as the physicochemical properties of bilayer foams.

## Methods

The Surface Evolver, written by Brakke^8^, is a computer program for finding a minimum energy structure under given constraints. A configuration of surfaces in Evolver has a finite element representation as a set of triangles, and begins as a crude finite element representation, which is then evolved by refining the triangulation and minimizing energy by moving vertices by gradient descent. The Surface Evolver works in dimensionless units, so the numerical values reported in this paper can be interpreted in any consistent system of units. Also, since the surface tension is the only energy present in the systems of this paper, the absolute value of the surface tension is immaterial.

A dry foam, in which the liquid fraction is zero, is the idealization that films have no thickness and the Plateau borders are regarded as curves. Real foams are wet, so their films have some thickness and have Plateau borders. The model used in this paper is an idealization to the extent that all the liquid is in the Plateau borders, and the dry part of the film has no thickness. The Surface Evolver has been adapted to calculate energies of dry foam structures and wet foam structures in research that has been reported^6^. In this paper we adopt such an application of the Evolver, and present some results. It is natural to begin at the extreme of dry foam, and compare honeycomb structure with Tóth structures on account of their configurations, and likely to be favored by increasing liquid fraction.

### Dry foams

**Figure 6:**
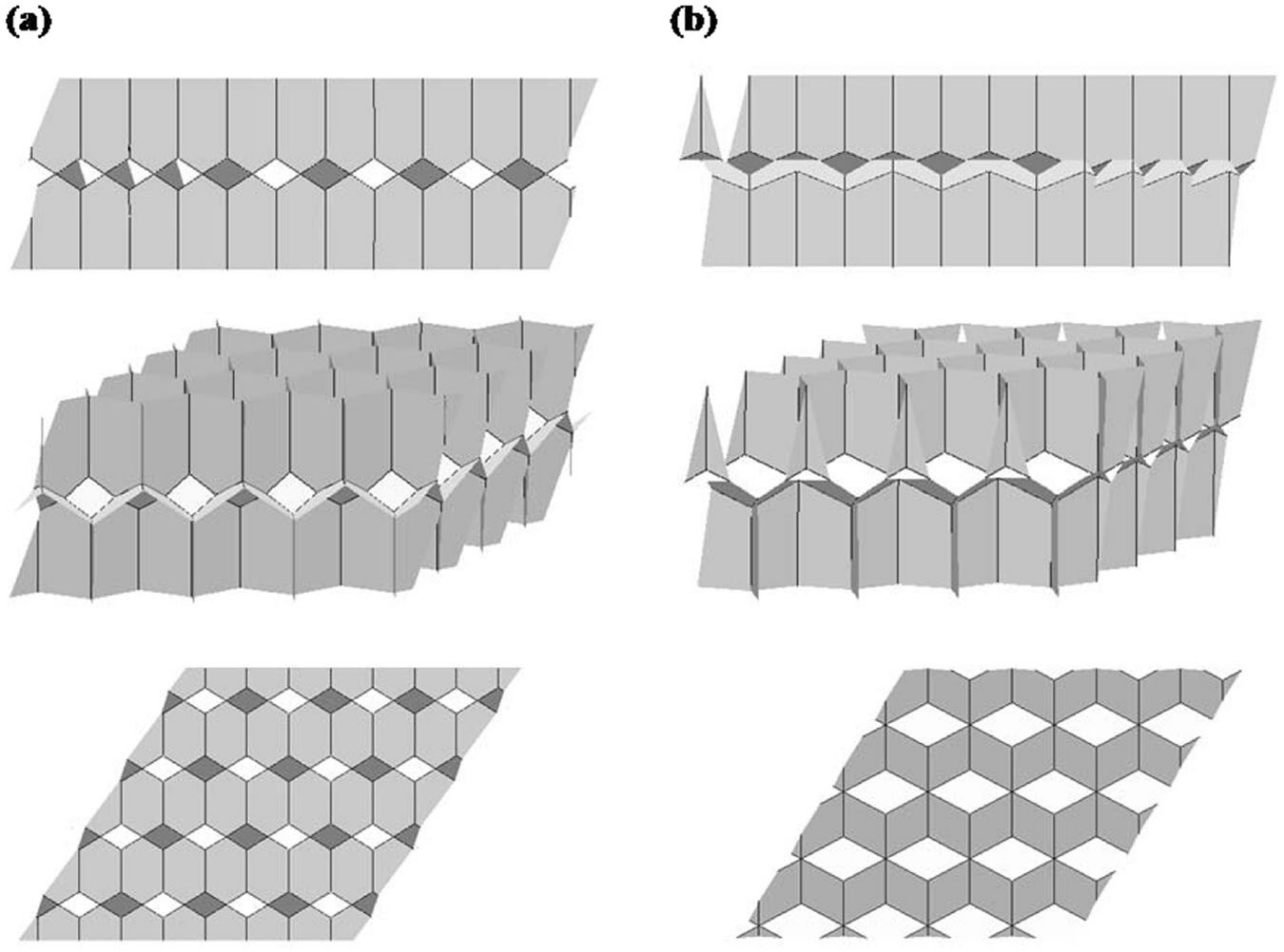
Surface Evolver simulations of two kinds of bilayer monodisperse systems for the dry foams: (**a**) Tóth structure. (**b**) Honeycomb structure. The upper-layer units of both these two structures are inverted above the lower-layer units. The white spots are not holes through the foam, but just a lighting effect on faces of a certain orientation.

To start with, as shown in Fig. 6, we simulate bilayer monodisperse dry foams between two flat horizontal plates with the configurations of the Tóth structure and the honeycomb structure. We form the models based on the principle of a foam with equal gas volume in each bubble, and each model has a structure such that the interfacial energy is a stable local minimum. To simulate space-filling foam, periodic boundary conditions are used, which Evolver calls the “torus model.” The Evolver models actually have only one upper and one lower cell; the illustrations show multiple copies of the basic unit. Each cell in the models has the same volume (the cell volume is √3 in this paper), so we can directly compare the equilibrium surface energies *E* of these two configurations^9, 10^.

### Wet foams

When liquid is introduced to a dry foam, it concentrates in the Plateau borders, since the high negative pressure there pulls the liquid from between the cell interface surfaces. The total free energy of this foam system consists of an interfacial contribution from the surface tension of the film interfaces. For the dry foams, the interfacial energy *E* is given by

1 $$E={T}_{f}{S}_{f}\cdot $$

where *T*_*f*_ is surface tension and *S*_*f*_ is the area of film interface per unit volume of foam. For the wet foams, the surface tension on a Plateau border is half that of a dry film (T_p_ = T_f_ /2), since a dry film is really a double layer^11^. So the interfacial energy *E* is given by

2 $$E={T}_{f}{S}_{f}+{T}_{p}{S}_{p}.$$

where *T*_*p*_ is Plateau border surface tension, and *S*_*p*_ is the area of the Plateau borders. The liquid fraction of a foam is designated by *φ*_*liq*_. For *φ*_*liq*_ > 0, Plateau’s rules do not strictly hold. In particular, more than four Plateau borders may meet in a single junction. In fact, the wet honeycomb structure has some eight-fold Plateau junctions, even at the driest volume fraction *φ*_*liq*_ = 0.001 considered in this paper.

For any given structure there is a maximum value of *φ*_*liq*_, defining the wet foam limit, beyond which the foam bubbles become separated. This has been called the “rigidity loss transition” by Bolton and Weaire^12^. Consideration of the range of *φ*_*liq*_ between 0 and the wet limit raises a number of questions^4^. What is the ideal bilayer monodisperse structure for each value of *φ*_*liq*_? How are the transition between Tóth structure and honeycomb structure dictated by instabilities and topological changes?

**Figure 7:**
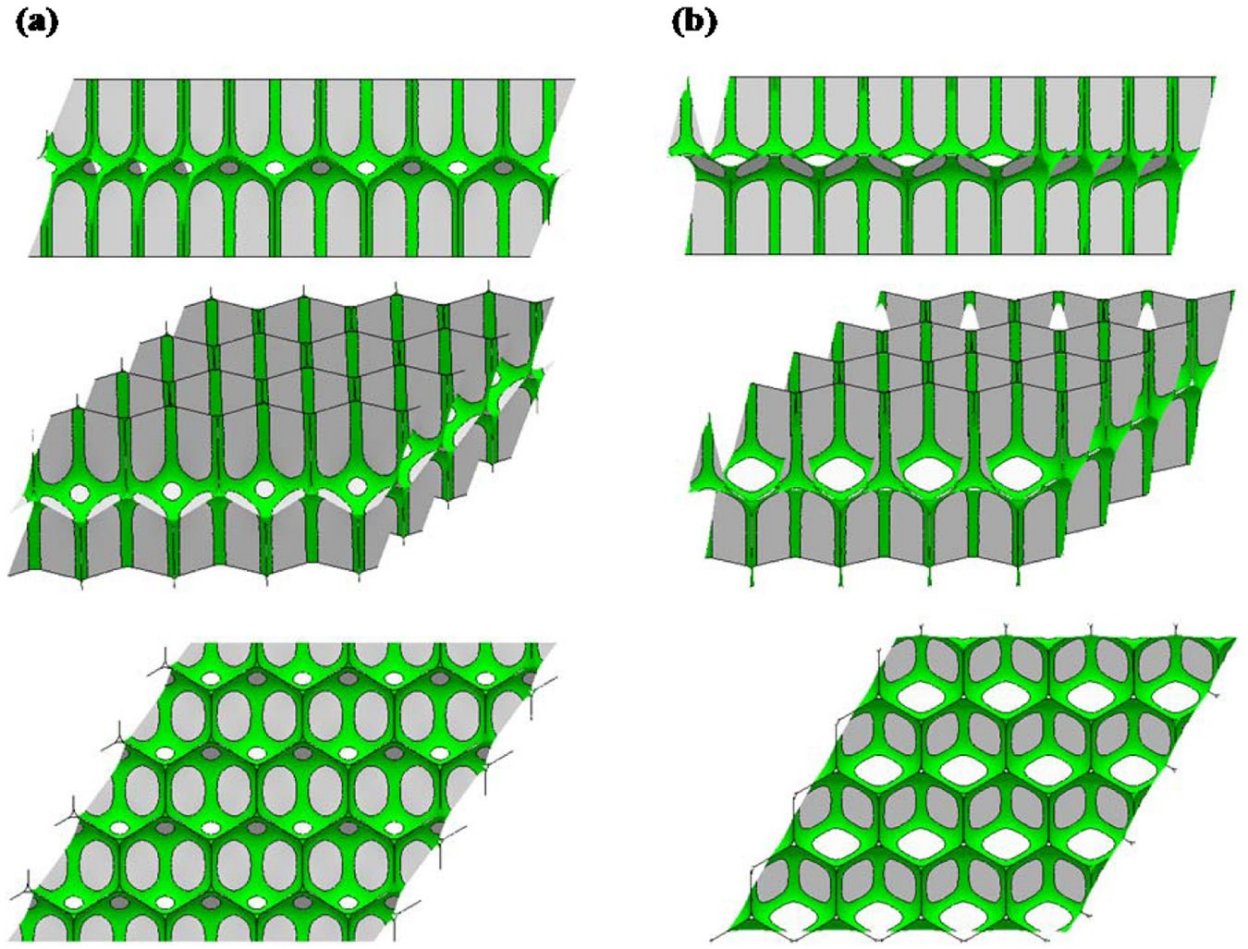
Surface Evolver simulations of two kinds of bilayer monodisperse systems for the wet foams: (**a**) Tóth structure. (**b**) Honeycomb structure. The green part represents Plateau borders; the grey part represents foam films. The liquid fraction is equal to the ratio of the green part volume the unit cell volume. The liquid fraction shown here is 2.00%. As in Fig. 2, the white ovals in figures are just some surfaces oriented for particularly bright lighting.

**Figure 8:**
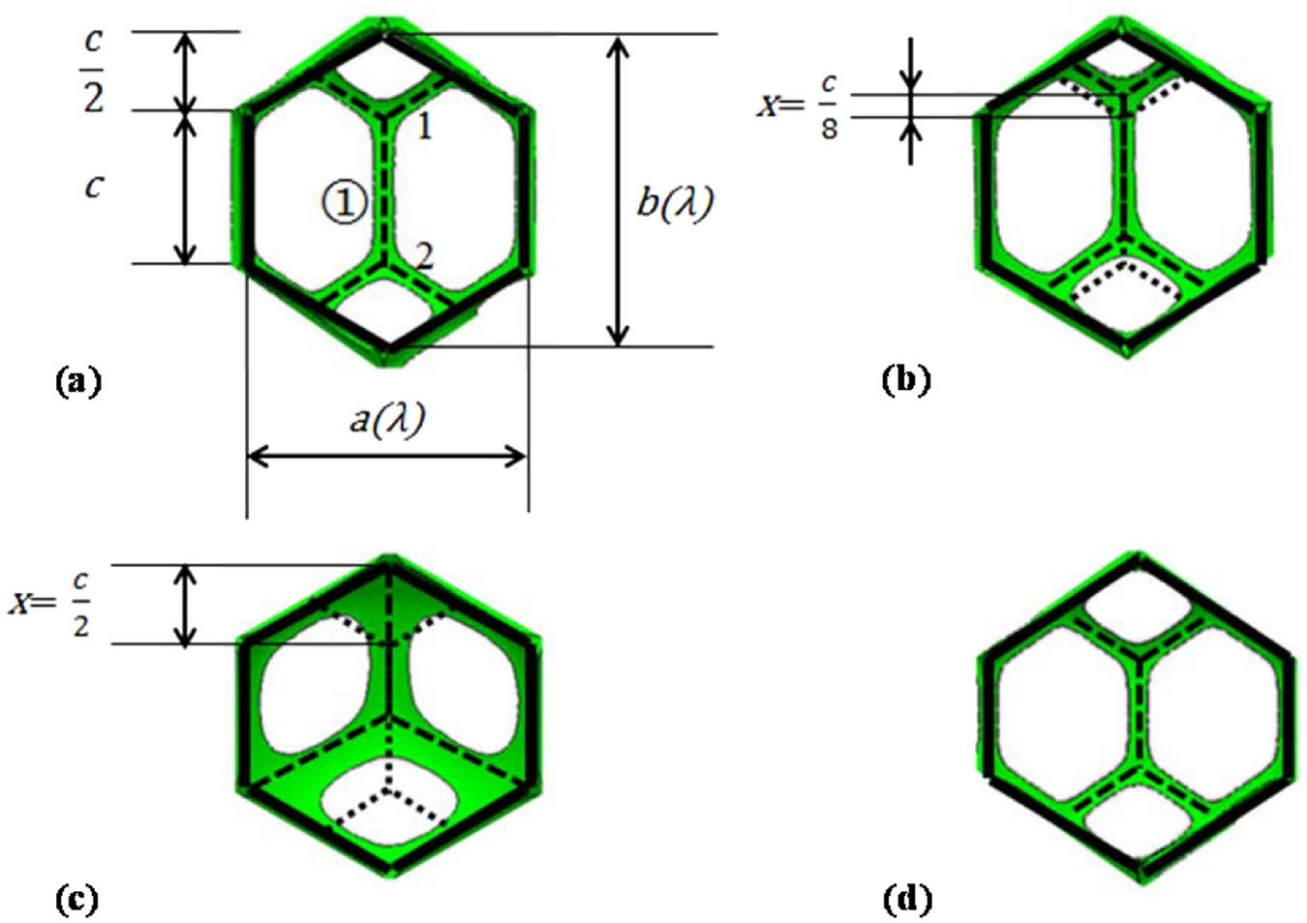
Sketches of top views of local configurations of Tóth, Tóth-like and honeycomb-like structures cell in the Evolver model: (**a**) Perfect Tóth structure. Here, *x* = 0, α = 0, λ = 1, *φ*_*liq*_ = 0.50%. (**b**) Tóth-like structure. Here, *x* = *c/8*, α = 0.25, λ = 1, *φ*_*liq*_ = 0.50%. (**c**) Honeycomb-like structure. Here, *x* = *c/2*, α = 1, λ = 1.05, *φ*_*liq*_ = 2.40%. (**d**) Tóth-like structure. Here, *x* = *0*, α = 0, λ = 1.1, *φ*_*liq*_ = 0.50%. In this figure, the foam cell/cell interface films are not shown, just the green Plateau border for clarity.

To address such questions, we used the Surface Evolver to get the equilibrium structure of bilayer monodisperse wet foams. To simulate the liquid content, we applied a Surface Evolver script that decorates dry bubble edges with Plateau borders of a certain size. Figures 7 and 8 present typical examples of equilibrium bilayer monodisperse foam structures generated in this way. We then controlled the liquid fraction to get different sizes of Plateau border, and evolved them with an evolution script so that the interfacial energy is minimal. The script evolves the surface at successive levels of refinement, until the energy change drops below 1e-8 for each gradient descent step. A second evolution script using different techniques was used to verify the results.

There are four geometric parameters in our models and they are independent variables. They are *φ*_*liq*_, *H*, α and λ. Here *φ*_*liq*_ is the fraction of liquid content in the unit cell. *H* is the distance between the horizontal boundaries, and *H* = 2.0 in Fig. 6 and Fig. 7. To see if there is an energy barrier to the Tóth structure moving to the honeycomb structure, we introduced a parameter α to index possible intermediate offsets between the two layers. We considered the Tóth structure α = 0 and honeycomb structure α = 1. Refering to Fig. 8, the specific definition of the parameter α is given by

3 $$\alpha =\frac{2}{c}x,$$

where *c* is the edge length of hexagonal contour. The length of vertical edge ➀ between vertex 1 and vertex 2 is equal to *c* and keeps constant when the value of α is changed. And *x* is the position (0 ≤ × ≤ c/2) of edge ➀ in the vertical direction, as Fig. 8 shows. When edge ➀ rises, all the lines inside the hexagonal contour move with the original angles preserved. Therefore, α = 1 when vertex 1 coincides with the contour vertex.

The aspect ratio parameter λ adjusts the horizontal torus periods^13^ (used for periodic boundary conditions in the Surface Evolver and to control the configuration of the unit cell), and can be used to find the periods with the lowest energy for the given height and liquid fraction. λ = 1 represents perfect hexagonal symmetry. It can be represented by

4 $$\{\begin{array}{c}a=\lambda \\ b=\frac{2}{\sqrt{3}\lambda }\cdot \end{array}$$

where *a*, *b* are the horizontal width and vertical length of the hexagon respectively, as shown in Fig. 8. Note that the area of the hexagon is constant when λ is changed.

In other words, it is a perfect Tóth structure only when α = 0 and λ = 1, as shown in Fig. 8a. Meanwhile, it is a perfect honeycomb structure only when α = 1 and λ = 1. In addition, to these two structures, we call structures Tóth-like and honeycomb-like when 0 < α < 1and λ ≠ 1 according to whether the Tóth structure’s extra interface film is present. For example, Fig. 8b and d both represent Tóth-like structures, and Fig. 8c is an example of honeycomb-like structure. Comparing the dashed lines inside the hexagonal contour of Fig. 8a–c, with the value of *x* increasing, α changes from 0 to 1. This corresponds to the configuration changing from Tóth structure to honeycomb structure in the Evolver model. The solid lines of the hexagonal contours of Fig. 8b–d have different aspect ratios in the horizontal direction of the hexagonal contour, for which the values of λ are 1, 1.05 and 1.1 respectively.

**Figure 9:**
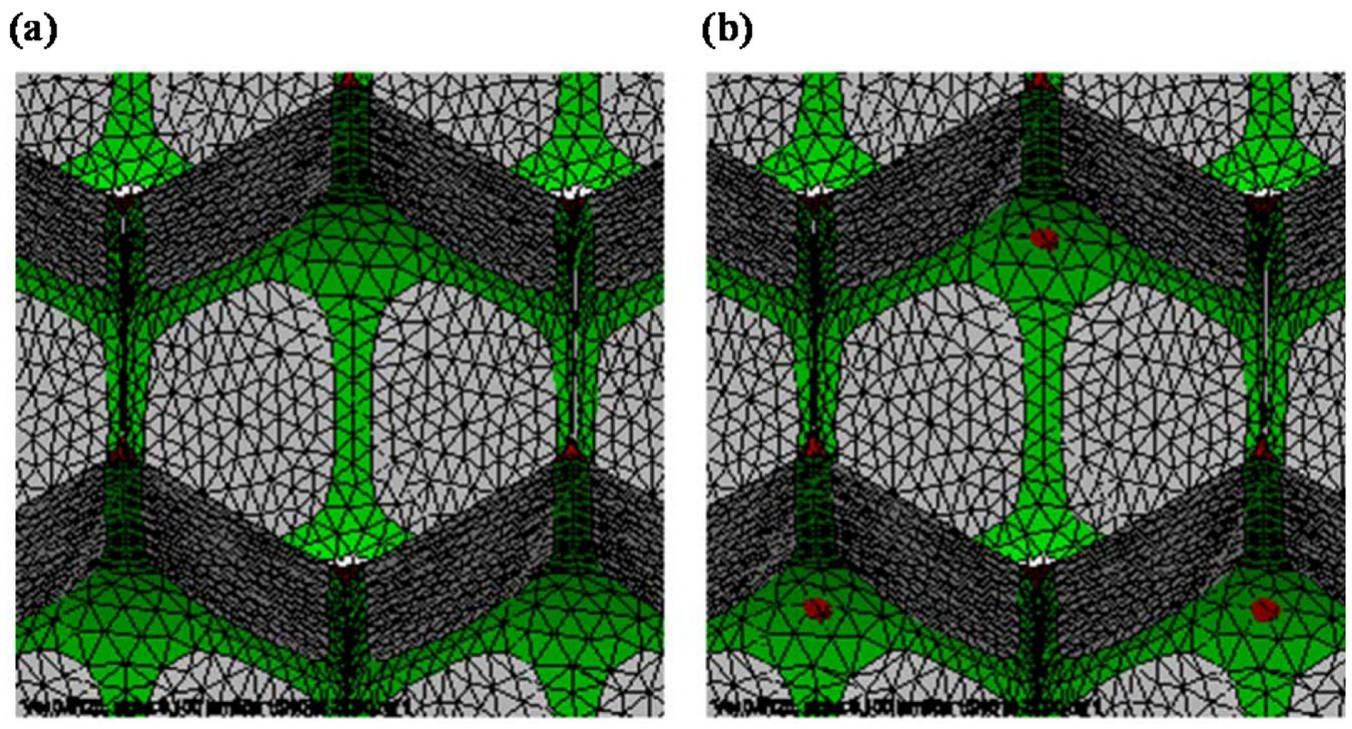
Overlap of cells in the transition from honeycomb to Tóth structure: (**a**) The cells just before overlap happens. (**b**) The cells just after overlap. The liquid side of the Plateau border surfaces is colored red. These images are from an early stage of evolution of the honeycomb structure for H = 2.0, *φ*_*liq*_ = 1.20%, α = 0.1, and λ = 1.0.

We have modeled and studied bilayer monodisperse foams under equilibrium conditions for liquid fractions in the range 0 < *φ*_*liq*_ < 3% for both structures. During evolution, it may happen that two cells on opposite sides of an eight-fold Plateau junction come together and form a new interface between the cells, while splitting the former Plateau junction into several four-fold junctions, as shown in Fig. 9. This happens when a honeycomb structure transforms into a Tóth structure. Evolver does not have built-in detection of surfaces running into each other, but our evolution scripts make periodic checks for such overlaps happening by a significant margin of the surfaces. It is difficult for Surface Evolver to deal with 3D topological transition dynamics^9^. In our simulation process, when the state is very near the transition point, faces of different cells overlap (as shown in Fig. 9) and the evolution is stopped. That means a structural transition occurred at this point in the corresponding physical system as reported in experiments^4^. Based on this method, the critical structural transition points were determined.

The reverse process of an interface disappearing and multiple Plateau junctions merging into one would happen if a Tóth structure were to change to a honeycomb structure, but such changes were not observed in the parameter range covered by this paper.

**Figure 10:**
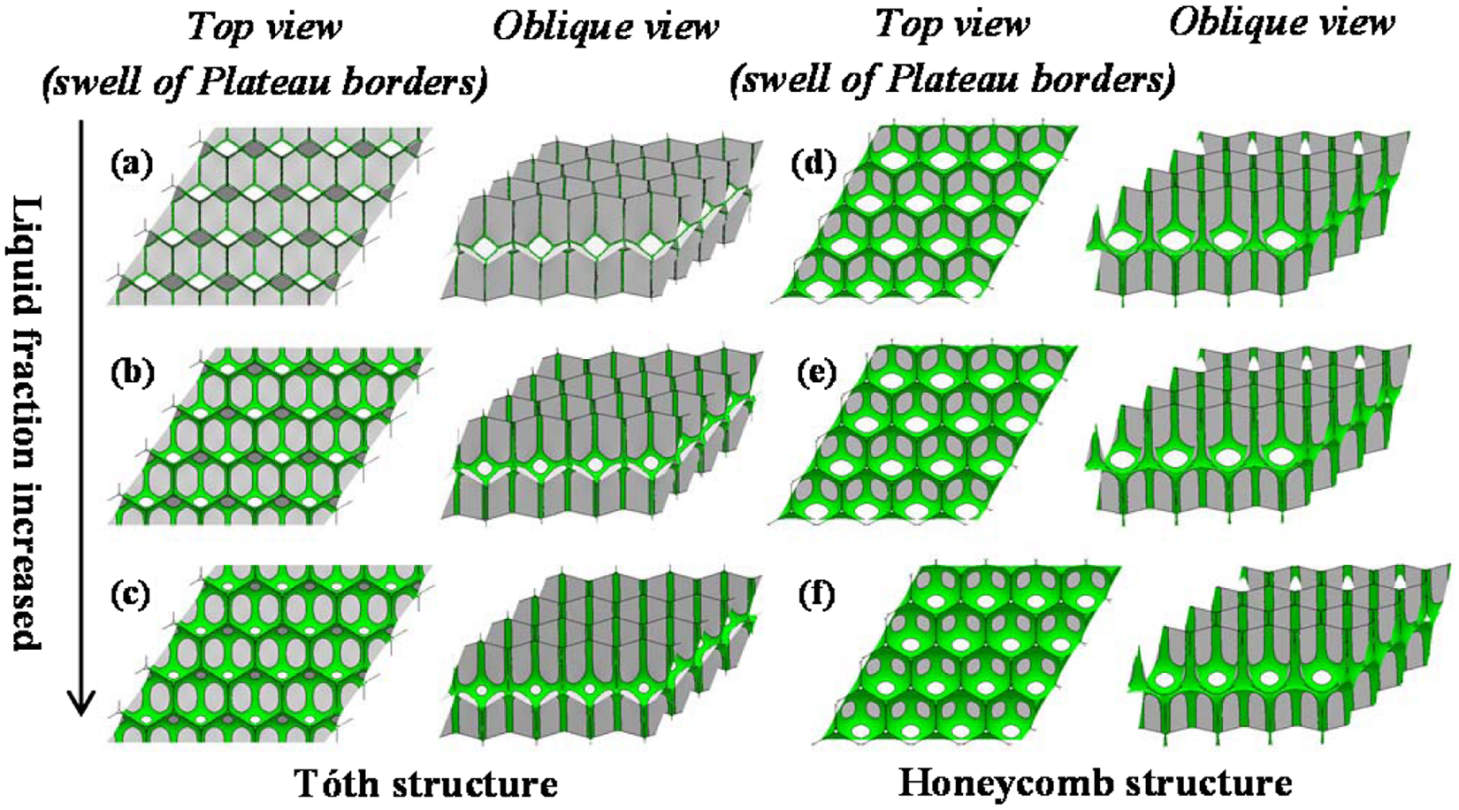
The configurations of two kinds of bilayer monodisperse foam structure with the variation of liquid fraction *φ*_*liq*_: Tóth structure: (**a**) 0.1%; (**b**) 1.0%; (**c**) 2.0%. Honeycomb structure: (**d**) 2.0%; (**e**) 3.0%; (**f**) 5.0%.

Figure 10 shows some different *φ*_*liq*_ examples of Tóth structure and honeycomb structure listed here. As the liquid fraction is increased, the foam becomes wetter, resulting in swelling of the Plateau borders, causing the foam to become less angular.

## References

[1] Mackenzie D (1999). Proving the Perfection of the Honeycomb. Science..

[2] Weaire, D. and Aste, T. *The pursuit of perfect packing*. (CRC Press, 2008).

[3] Tóth LF (1964). What the bees know and what they do not know. Bull Am Math Soc.

[4] Weaire D, Phelan R (1994). Optimal design of honeycombs. Nature..

[5] Phelan R, Weaire D, Brakke KA (1995). Computation of equilibrium foam structures using the Surface Evolver. Experimental Mathematics..

[6] Kraynik AM, Reinelt DA (1996). Linear elastic behavior of dry soap foams. Journal of Colloid and Interface Science.

[7] Plateau JAF (1873). Statique expérimentale et théorique des liquides soumis aux seules forces moléculaires. Gauthier-Villars.

[8] Brakke, K. A. The Surface Evolver, *Experimental Mathematics*. **1**, 141–165(1992). The software is available from http://www.susqu.edu/brakke/evolver (2013).

[9] Hutzler S, Saadatfar M, Van DNA (2008). The dynamics of a topological change in a system of soap films. Colloids and Surfaces A:Physicochemical and Engineering Aspects..

[10] Höhler R, Sang YYC, Lorenceau E (2008). Osmotic pressure and structures of monodisperse ordered foam. Langmuir..

[11] Brakke KA (2005). Instability of the wet cube cone soap film. Colloids and Surfaces A:Physicochemical and Engineering Aspects..

[12] Bolton F, Weaire D (1990). Rigidity loss transition in a disordered 2D froth. Physical review letters.

[13] Brakke, K. A. *Surface evolver manual*. (Susquehanna University, 1994).

